# Development and validation of a prostate cancer risk prediction model for the elevated PSA population

**DOI:** 10.3389/fonc.2025.1599266

**Published:** 2025-09-15

**Authors:** Junhui Wu, Xiaodong Jin, Jiali Li, Lingqian Zhao, Chenkai Zhao, Nengfeng Yu, Yubing Li, Jiasheng Yan, Junlong Wang, Fei Yang, Wenhao Zhang

**Affiliations:** ^1^ Department of Urology, The First Affiliated Hospital of Zhejiang Chinese Medical University (Zhejiang Provincial Hospital of Chinese Medicine), Hangzhou, Zhejiang, China; ^2^ Center for Reproductive Medicine, Department of Gynecology, Zhejiang Provincial People’s Hospital (Affiliated People’s Hospital, Hangzhou Medical College), Hangzhou, Zhejiang, China

**Keywords:** prostate biopsy, prostate-specific antigen, prostate cancer, prediction model, risk stratification, neutrophil-to-lymphocyte ratio, LASSO

## Abstract

**Introduction:**

To develop and validate a dynamic clinical prediction model integrating prostate-specific antigen (PSA) and peripheral blood biomarkers for distinguishing benign from malignant prostate diseases in patients with elevated PSA levels.

**Methods:**

A retrospective study was conducted of clinicopathological data and preoperative blood specimen information of patients who underwent ultrasound-guided prostate biopsy in The First Affiliated Hospital of Zhejiang Chinese Medical University due to elevated PSA between January 2018 and November 2024.Univariate analysis, Least Absolute Shrinkage and Selection Operator regression, and multifactorial logistic regression analysis were utilized to identify independent risk factors associated with benign or malignant prostate disease in patients with elevated PSA (PSA > 4.0ng/ml). The construction of a clinical prediction model was then undertaken, with the subsequent calibration and integration into a network calculator.

**Results:**

A total of 529 patients were included based on predefined inclusion and exclusion criteria, comprising 268 (50.7%) with benign pathology and 261 (49.3%) with malignancy. After analysis, independent risk factors associated with benign or malignant prostatic diseases in patients with elevated PSA levels were identified, including PSA, white blood cell, neutrophil-to-lymphocyte ratio, lymphocyte-to-monocyte ratio, eosinophil count, basophil count, and serum albumin. Utilizing these independent risk factors, a clinical prediction model for the risk of PSA-elevated prostate benign-malignant disease was constructed, yielding an area under the curve of 0.906, a predictive model specificity of 77.6%, and a sensitivity of 95%. The calibration curve and clinical decision curve indicated that the model exhibited superior calibration ability. A dynamic prediction model was formulated based on the clinical prediction model integrated into a network calculator.

**Conclusion:**

This study establishes a non-invasive prediction model integrating PSA and peripheral blood biomarkers, providing a clinically practical tool for risk stratification in patients with elevated PSA levels.

## 1 Introduction

Prostate cancer (PCa) is the most prevalent malignancy in the male genitourinary system. According to the Global Cancer Statistics 2022, PCa was estimated to account for approximately 1.47 million new cases and 397,000 deaths globally, representing 7.3% of all incident cancer cases and 4.1% of cancer-related mortality in 2022, underscoring its significant burden on men’s health (1). Prostate-specific antigen (PSA) remains the most widely used biomarker for PCa screening (2). However, elevated PSA levels are not specific to malignancy and may also occur in benign prostatic conditions such as prostatitis and benign prostatic hyperplasia (BPH) (3). Therefore, magnetic resonance imaging (MRI) and ultrasound-guided prostate biopsy remain the gold standard for definitive diagnosis. Prostate biopsy is an invasive and complex procedure that demands significant operator expertise. Moreover, its potential for false-negative results and patient discomfort contribute to its limited acceptance among some individuals in clinical practice. Gilbert et al. analyzed 36,316 prostate biopsy cases and found a positivity rate of 30.08% when PSA levels ranged between 4–10 ng/mL. However, even when PSA exceeded 10 ng/mL, the positivity rate only increased to 40.58% (4). As a result, the proper selection of candidates for prostate biopsy is crucial for both preventing missed diagnoses and minimizing unnecessary invasive procedures. According to the 2025 European Association of Urology guidelines, men with elevated PSA levels should undergo further evaluation, including PSA density (PSAD), digital rectal examination, and multiparametric MRI before deciding on biopsy. In patients with PI-RADS ≤2 and PSAD <0.15–0.20 ng/mL/cm³, prostate biopsy can generally be deferred. For PI-RADS 3 lesions, a PSAD <0.10 ng/mL/cm³ may be considered as a criterion to delay biopsy in selected cases, particularly when clinical suspicion remains low (5). Nevertheless, the interpretation of PI-RADS is still influenced by the experience of the radiologist and image quality, while PSAD values are affected by errors in prostate volume estimation. These factors may lead to variability and inconsistency in screening results. Consequently, developing additional diagnostic methods to assist in distinguishing benign from malignant prostate conditions in patients with elevated PSA levels remains a critical clinical challenge.

To address this issue, novel biomarkers such as the Prostate Health Index (PHI), SelectMDx, 4Kscore, and ExoDx Prostate IntelliScore (EPI) have been developed. These tools integrate multiple biomarkers to improve diagnostic accuracy for distinguishing benign from malignant prostate conditions (6). However, these models also present certain limitations. Firstly, most models rely on expensive equipment and complex technologies, which restrict their widespread use in low-resource settings (7). Secondly, due to racial and clinical differences, the predictive accuracy of these models in Eastern Asian populations may differ from that in Western populations, leading to limited applicability and effectiveness in Asian populations (8). Finally, although these models have demonstrated good performance in patients within the PSA “gray zone”, the negative predictive values vary substantially across studies, suggesting that predictive accuracy may fluctuate in populations with lower PSA levels or reduced tumor burden (9).

In recent years, peripheral blood markers have been widely used in clinical research in oncology. Blood routine indicators such as white blood cell count, lymphocyte count and platelet count can reveal the immune function and inflammation level of cancer patients, and show great potential in the early diagnosis, prognosis assessment and treatment monitoring of tumors (10–12). Furthermore, serum biochemical markers including calcium, phosphorus, alkaline phosphatase, and lipid metabolism indicators are routinely used to assess tumor progression or metastasis (13, 14). Although peripheral blood markers have been widely applied in cancer risk prediction studies, their predictive performance varies considerably across different populations, cancer types, and research settings. First, these markers generally lack organ specificity and are susceptible to various non-malignant influences, such as low-grade chronic inflammation, baseline metabolic differences, and dietary patterns (15). Second, certain biochemical indicators, such as lipid profiles, calcium, and phosphorus, may exhibit notable short-term variability, which could compromise the stability and generalizability of predictive models (16). Moreover, the cut-off values used for these markers differ significantly among studies, and no standardized threshold has been established, limiting the applicability of such models across diverse populations (17). Therefore, while peripheral blood markers hold promise in cancer risk prediction, their clinical utility requires further validation and optimization under standardized conditions and within specific population contexts. This study systematically evaluated multiple peripheral blood biomarkers in individuals with elevated PSA levels and developed a PCa risk prediction model based on a Chinese male population. The model aims to improve the accuracy of early diagnosis, reduce unnecessary invasive procedures, and ensure both clinical feasibility and regional applicability, thereby serving as a valuable supplement to existing risk stratification strategies.

## 2 Materials and methods

### 2.1 Study population

Clinical and pathological data were retrospectively analyzed from patients who underwent ultrasound-guided prostate biopsy at The First Affiliated Hospital of Zhejiang Chinese Medical University between January 2018 and November 2024 due to elevated PSA levels. Participants were stratified into benign or malignant groups based on post-biopsy histopathological results. Inclusion criteria: (1) PSA>4ng/ml; (2) underwent prostate aspiration biopsy. Exclusion criteria: (1) history of other malignancies or prior cancer-related therapies; (2) hematologic disorders; (3) autoimmune diseases; (4) acute or chronic inflammatory diseases, or other diseases affecting routine blood tests or biochemical tests; (5) Incomplete clinical or pathological records.

### 2.2 Clinical data extraction

General clinical information of enrolled patients was collected, including age, height, weight, and body mass index (BMI) was calculated. Fasting peripheral blood samples were collected within one week prior to biopsy for automated complete blood count analysis. All tests were conducted in the clinical laboratory of our hospital using standardized procedures and uniform automated analyzers. All reagents were supplied by the same manufacturer, with batch numbers recorded for traceability. Quality control comparisons were performed upon batch replacement to minimize potential batch-to-batch variability. Manually recorded parameters included: absolute counts of white blood cells (WBC), lymphocytes (LYM), monocytes (MONO), neutrophils (NEUT), platelets (PLT), eosinophils (EOS), basophils (BASO), and red blood cells (RBC); mean corpuscular volume (MCV); hemoglobin (HB); serum calcium (Ca); serum phosphorus (P); total cholesterol (TC); triglycerides (TG); high-density lipoprotein (HDL); low-density lipoprotein (LDL); albumin (ALB); and alkaline phosphatase (ALP). Derived ratios were calculated: neutrophil-to-lymphocyte ratio (NLR), platelet-to-lymphocyte ratio (PLR), lymphocyte-to-monocyte ratio (LMR), and systemic immune-inflammation index (SII = [PLT × NEUT]/LYM).

### 2.3 Statistical analysis

Continuous variables are presented as mean ± standard deviation (± S), while categorical variables are expressed as frequencies and percentages. Statistical analyses were performed using SPSS (v26.0) and R Studio (v1.4) with relevant packages. Independent samples t-tests were applied to compare continuous variables between groups, and variables with statistically significant differences (*P <*0.05) were selected as candidate risk factors. The relevant factors were used in the least absolute shrinkage and selection operator (LASSO) regression algorithm to create a dummy variable. The appropriate adjustment parameter (*λ*) for the LASSO regression was determined using cross-validation. The model fitting effect was evaluated at different *λ* values, and the maximum *λ* value was selected as the value of “lambda. 1se” when the average error was within one standard deviation. That is to say, the risk factors with non-zero regression coefficients when *λ* was “lambda. 1se” were screened. A subsequent logistic regression analysis was performed on the screened risk factors to further identify factors associated with risk stratification. The odds ratio (OR) and 95% confidence interval (95% CI) were determined to identify independent risk factors for the malignancy of the prostate with elevated PSA.

### 2.4 Model construction and verification

A clinical prediction model was constructed to evaluate the predictive efficacy and discriminant ability of the model using the receiver operating characteristic (ROC) curve, area under the curve (AUC) value, 95%CI, Youden index, sensitivity and specificity. The established clinical prediction model was internally validated using the Bootstrap method with 1,000 samples, and a calibration plot was drawn to verify the consistency between the model prediction results and the actual results. A decision curve analysis (DCA) was drawn to evaluate the clinical net benefit rate of the model in the modeling group. The above methods were used to verify the calibration ability of the model. Based on the establishment and verification of the clinical prediction model, an online calculator was launched using the R language to establish a dynamic prediction model.

## 3 Results

### 3.1 Sample size estimation and study population characteristics

This study involved a predictive model for categorical outcomes, and the required sample size was estimated using the formula: n = EPV × k/p, where EPV refers to the minimum number of outcome events required per predictor, typically ranging from 10~20. An EPV value of 20 was adopted in this study. k represents the number of predictors, which was determined to be 7 based on preliminary experiments. p refers to the incidence of malignant prostate tumors, reported in previous studies as 30.08%. Thus, the minimum required sample size was calculated as n = 20 × 7/0.3008 ≈ 466 cases. Based on the inclusion and exclusion criteria, 529 patients undergoing prostate biopsy were enrolled in the study. Pathological results confirmed 268 benign cases (50.7%) and 261 malignant cases (49.3%). General patient information is summarized in **Table 1**.

**Table 1:** Baseline characteristics of the study population.

Characteristics		Total	Benign	Malignancy
		529	268	261
Gleason Score	6			19
	7			149
	8			31
	9			60
	10			2
Age		69.9 ± 7.7	69.5 ± 8.5	70.3 ± 6.7
Height		160.1 ± 5.5	168.5 ± 5.4	167.6 ± 5.6
Weight		67.1 ± 9.2	67.5 ± 9.0	66.6 ± 9.4
BMI		23.7 ± 2.8	23.8 ± 2.8	23.7 ± 2.9
fPSA		2.1 ± 2.0	2.1 ± 2.1	2.1 ± 1.9
PSA*		14.1 ± 15.8	10.1 ± 6.6	18.2 ± 20.7
WBC*		5.9 ± 1.6	6.1 ± 1.7	5.8 ± 1.5
NEUT		3.8 ± 1.5	3.9 ± 1.5	3.7 ± 1.4
LYM		1.5 ± 0.5	1.6 ± 0.5	1.4 ± 0.6
MONO		0.5 ± 0.2	0.5 ± 0.2	0.4 ± 0.2
NLR*		2.0 ± 2.1	1.1 ± 2.2	2.8 ± 1.6
LMR*		2.4 ± 2.2	1.1 ± 1.9	3.6 ± 1.8
PLR*		134.4 ± 847.8	51.3 ± 86.5	219.9 ± 1199.0
SII*		413.3 ± 438.1	272.9 ± 437.9	557.5 ± 389.4
EOS*		0.2 ± 0.1	0.1 ± 0.1	0.2 ± 0.1
BASO*		0.03 ± 0.02	0.03 ± 0.02	0.03 ± 0.02
RBC*		4.5 ± 0.6	4.6 ± 0.5	4.4 ± 0.6
MCV		91.5 ± 6.5	91.5 ± 5.1	91.5 ± 7.8
HB*		138.3 ± 15.5	139.8 ± 15.6	136.7 ± 15.3
PLT		199.8 ± 65.2	204.5 ± 68.1	195.0 ± 62.0
Ca*		2.3 ± 0.2	2.3 ± 0.3	2.3 ± 0.1
P		1.1 ± 0.3	1.0 ± 0.2	1.1 ± 0.3
TC		4.4 ± 0.9	4.4 ± 0.8	4.4 ± 1.0
TG		1.6 ± 0.9	1.6 ± 1.0	1.6 ± 0.9
HDL		1.2 ± 0.3	1.2 ± 0.3	1.2 ± 0.3
LDL		2.4 ± 0.7	2.3 ± 0.6	2.4 ± 0.7
ALB*		39.4 ± 4.6	40.7 ± 4.5	38.2 ± 4.5
ALP		78.5 ± 31.9	80.5 ± 39.3	76.5 ± 21.7

This table summarizes the baseline characteristics of all enrolled patients who underwent prostate biopsy, categorized into benign and malignant groups based on final pathological results. Variables marked with an asterisk (*) indicate statistically significant differences between the benign and malignant groups (*P* < 0.05), with specific *P*-values detailed in the univariate analysis column of **Table 2**.

### 3.2 Results of LASSO regression and logistic regression analysis

The results of the univariate t-test analysis demonstrated that PSA, WBC, NLR, LMR, PLR, SII, EOS, BASO, RBC, HB, Ca, and ALB were significantly associated with the discrimination between benign and malignant prostate conditions in the context of elevated PSA levels (*P <*0.05). While Age, height, weight, BMI, free PSA (fPSA), NEUT, LYM, MONO, MCV, PLT, P, TC, TG, HDL, LDL, and ALP showed no statistical significance (**Table 2**).

**Table 2:** Results of univariate and logistic analysis of the benign and malignant groups of prostate biopsies.

Characteristics	*P* (univariate analysis)	*P* (multivariate analysis)	B	OR	95% CI
Age	0.261				
Height	0.429				
Weight	0.144				
BMI	0.301				
fPSA	0.806				
PSA	<0.001	0.028	0.078	1.081	1.047-1.117
WBC	0.019	<0.001	-0.380	0.684	0.572-0.818
NEUT	0.093				
LYM	0.063				
MONO	0.341				
NLR	<0.001	<0.001	0.464	1.591	1.356-1.866
LMR	<0.001	<0.001	0.687	1.988	1.709-2.314
PLR	0.022	0.057			
SII	<0.001	0.896			
EOS	0.027	0.015	2.851	17.302	1.758-170.238
BASO	<0.001	0.030	14.467	1918955.36	4.079-9.027E+11
RBC	0.003	0.804			
MCV	0.959				
HB	0.019	0.367			
PLT	0.096				
Ca	0.035	0.860			
P	0.097				
TC	0.534				
TG	0.695				
HDL	0.762				
LDL	0.280				
ALB	<0.001	<0.001	-0.116	0.891	0.839-0.946
ALP	0.151				

This table presents P-values from univariate and multivariate analyses, regression coefficients (B), OR, and 95% CI for each variable. Variables with statistically significant associations in the multivariate model (*P* < 0.05) included PSA, WBC, NLR, LMR, EOS, BASO and ALB.

**Figure 1:**
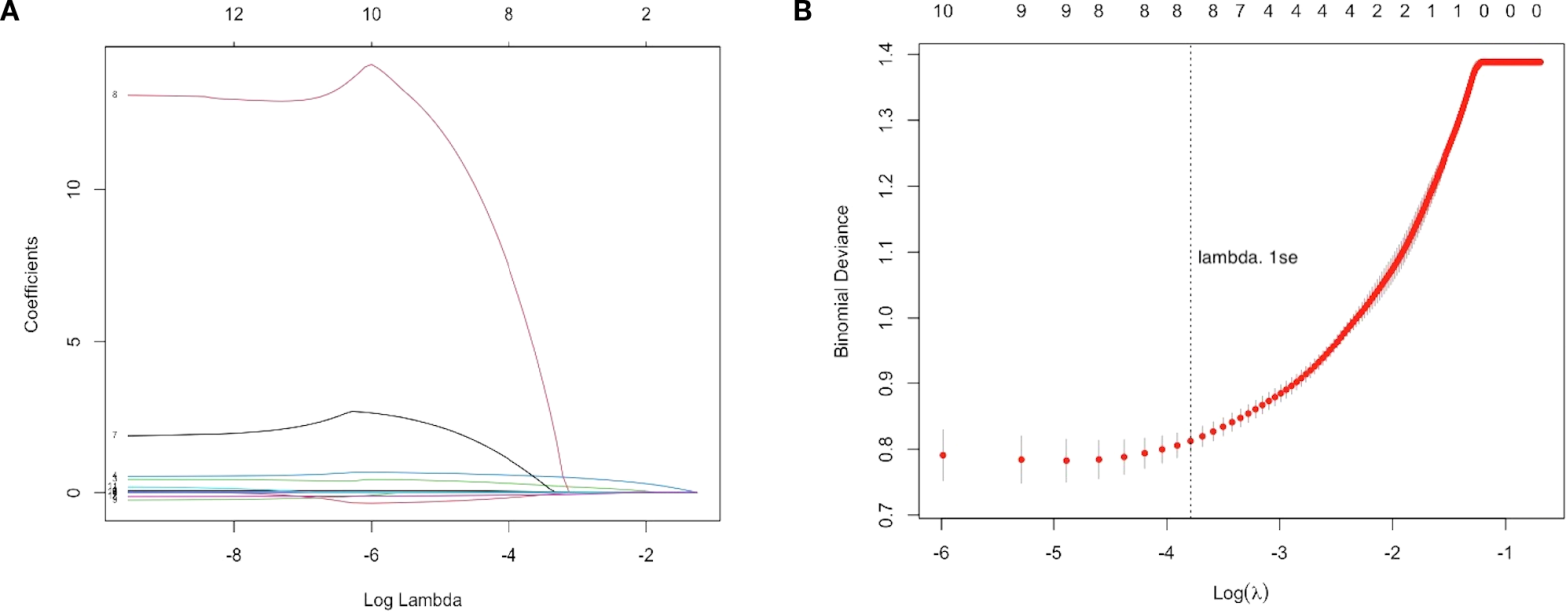
LASSO regression analysis for feature selection. **(A)** LASSO coefficient path plot with the x-axis representing the log-transformed regularization parameter log(λ) and the y-axis showing the regression coefficients of each variable. As λ increases, most coefficients shrink toward zero, indicating reduced predictive contribution. **(B)** Three-fold cross-validation curve with the x-axis showing log(λ) and the y-axis showing the binomial deviance. Red dots represent the mean deviance for each λ, with error bars indicating the standard error. The vertical dotted line marks the optimal λ selected using the 1-standard error rule (lambda.1se), which achieves a parsimonious model with good predictive performance. Eight variables with non-zero coefficients were retained for model construction.

The LASSO regression analysis set the cross-validation seed value to 123, and the optimal adjustment parameter λ value was 0.000072.The relationship between coefficients and *λ* values is shown in **Figure 1A**. Using 3-fold cross-validation to evaluate the model, **Figure 1B** displays the cross-validation curve illustrating the change in deviance with *λ* values. The “lambda. 1se” (the largest *λ* value within one standard error of the minimum mean error) was selected, identifying 8 variables with non-zero regression coefficients at this *λ* value: PSA, WBC, NLR, LMR, PLR, EOS, BASO, and ALB.

Subsequent multivariable logistic regression analysis identified eight independent risk factors for prostate malignancy associated with elevated PSA levels: PSA (*P* = 0.028, OR: 1.081, 95% CI: 1.047-1.117), WBC (*P <*0.001, OR: 0.684, 95% CI: 0.572-0.818), NLR (*P <*0.001, OR: 0.464, and 95% CI: 1.356-1.866), LMR (*P <*0.001, OR: 0.687, 95% CI: 1.709-2.314), EOS (*P* = 0.015, OR: 17.302, 95% CI: 1.758-170.238), BASO (*P* = 0.030, OR: 1918955.36, 95% CI: 4.079-9.027E+11), and ALB (*P <*0.001, OR: 0.891, 95% CI: 0.839-0.946).

### 3.3 Model construction and evaluation

**Figure 2:**
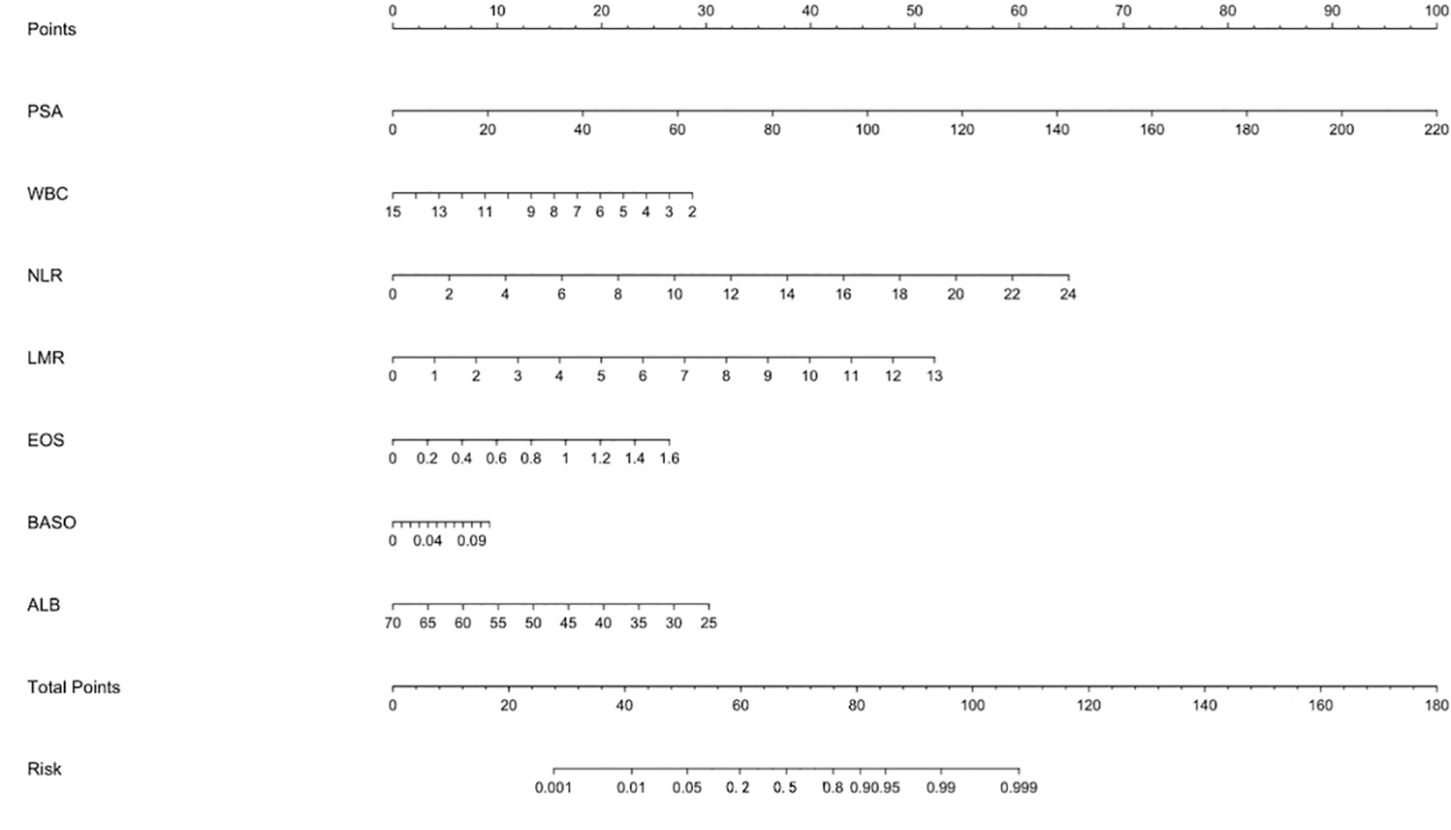
Nomogram for predicting the risk of malignant prostate disease in patients with elevated PSA. The nomogram was developed based on a multivariate logistic regression model, incorporating seven variables: PSA, WBC, NLR, LMR, EOS, BASO, and ALB. For each patient, a point is assigned for each variable value by projecting upward to the “Points” scale. The sum of all points gives a “Total Points” score, which corresponds to the predicted probability of malignancy at the bottom “Risk” scale.

**Figure 3:**
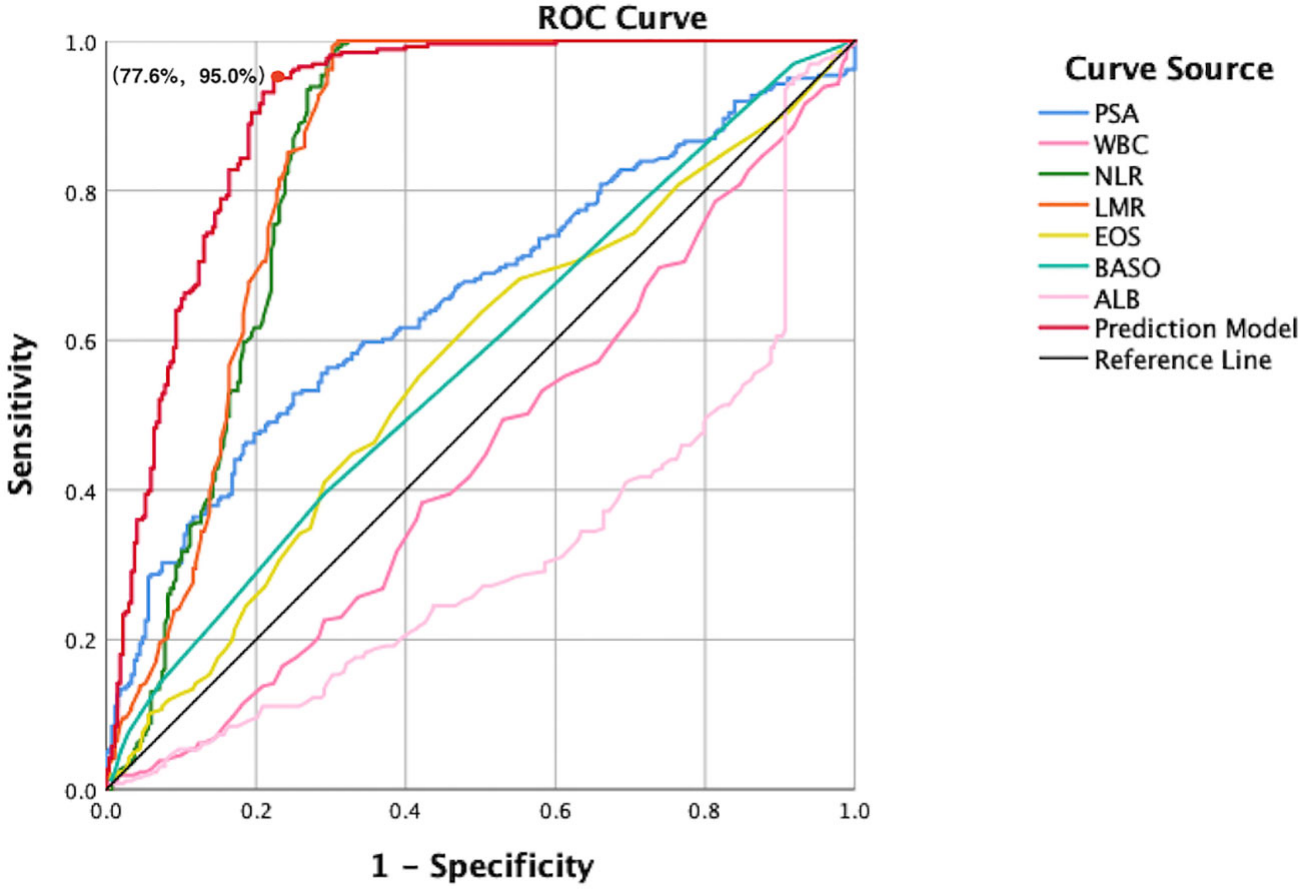
ROC curves comparing individual predictors and the combined prediction model. This figure presents the diagnostic performance of PSA, WBC, NLR, LMR, EOS, BASO, ALB, and the integrated model in differentiating benign from malignant prostate disease. The x-axis represents the false positive rate (1-specificity), and the y-axis represents the true positive rate (sensitivity). The red curve indicates the combined prediction model, which achieves the highest AUC, outperforming all single predictors. The diagonal reference line represents the performance of a non-informative classifier, and serves as a baseline for comparison. At the point of maximum Youden index, the model achieved a specificity of 77.6% and a sensitivity of 95.0%, indicating excellent discriminative capacity.

**Figure 4:**
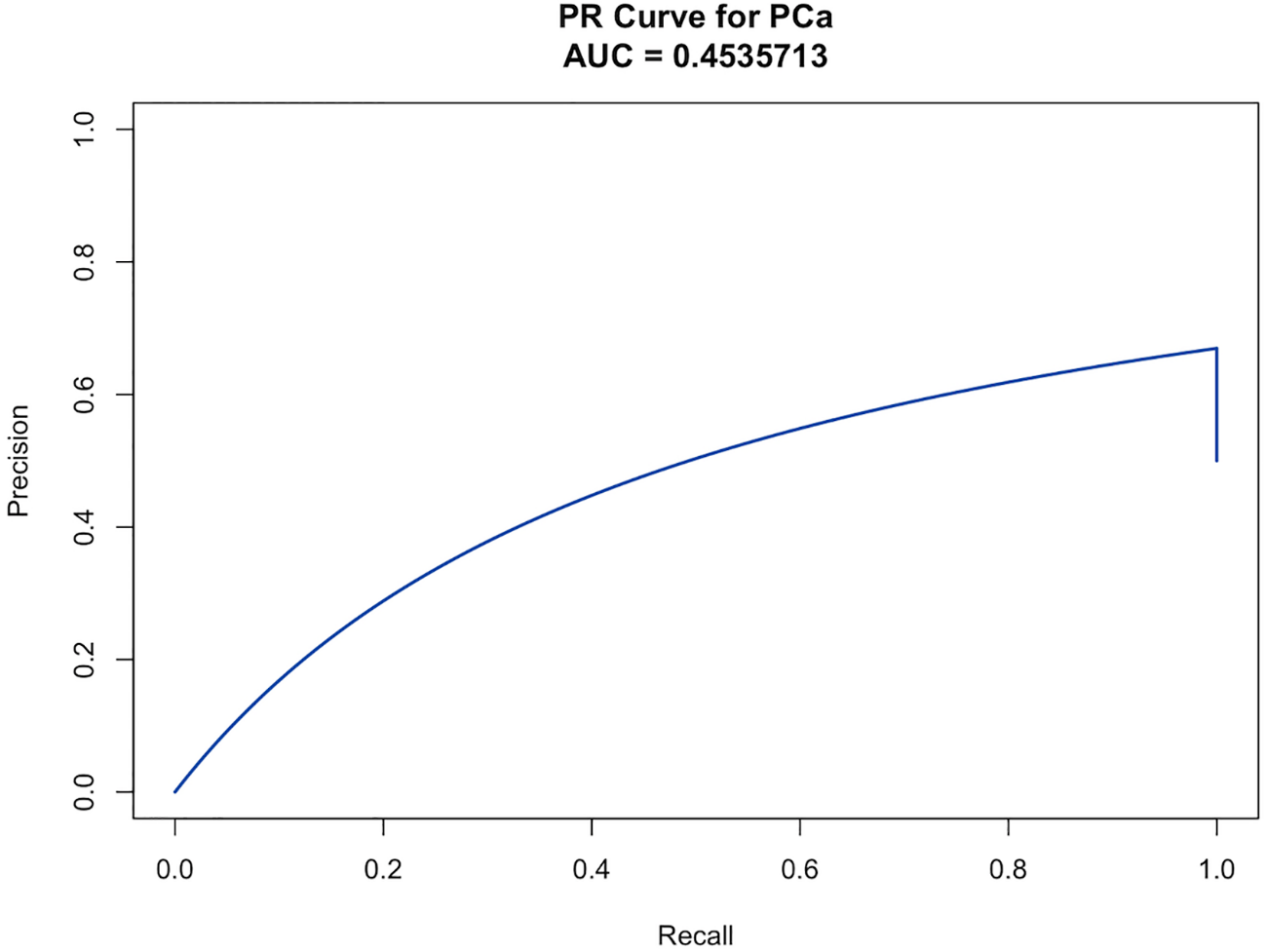
The PR curve illustrates the model’s ability to identify malignant prostate cases among patients with elevated PSA, based on the trade-off between precision and recall across different thresholds. The AP was 0.454, reflecting the model’s predictive performance in detecting positive cases.

A clinical prediction model for distinguishing benign from malignant prostate disease in patients with elevated PSA was established by integrating independent risk factors identified through logistic regression analysis: PSA, WBC, NLR, LMR, EOS, BASO, and ALB (**Figure 2**). ROC curve analysis demonstrated an AUC of 0.906 (95% CI: 0.8791–0.9323), with sensitivity and specificity of 95.0% and 77.6%, respectively, at the maximum Youden index (**Figure 3**). The precision–recall (PR) curve yielded an average precision (AP) of 0.45 (**Figure 4**), indicating that the model possesses a certain degree of discriminative ability in identifying positive cases.

**Figure 5:**
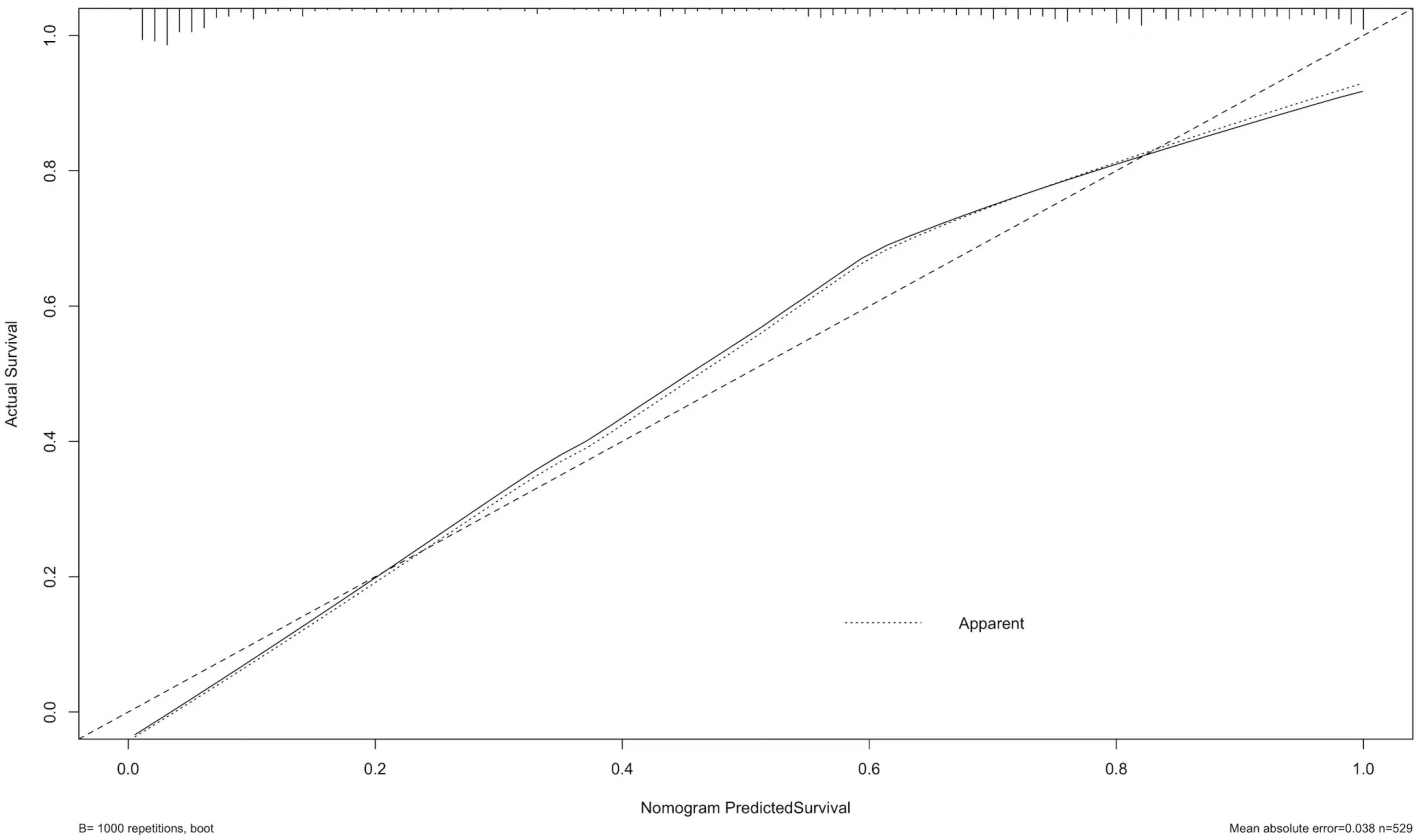
The calibration curve assesses the agreement between predicted probabilities and actual outcomes. The dashed line represents perfect prediction, while the solid line shows the model’s calibration based on 1,000 bootstrap resamples. The result indicates good consistency between predicted and observed outcomes, with a mean absolute error of 0.038.

**Figure 6:**
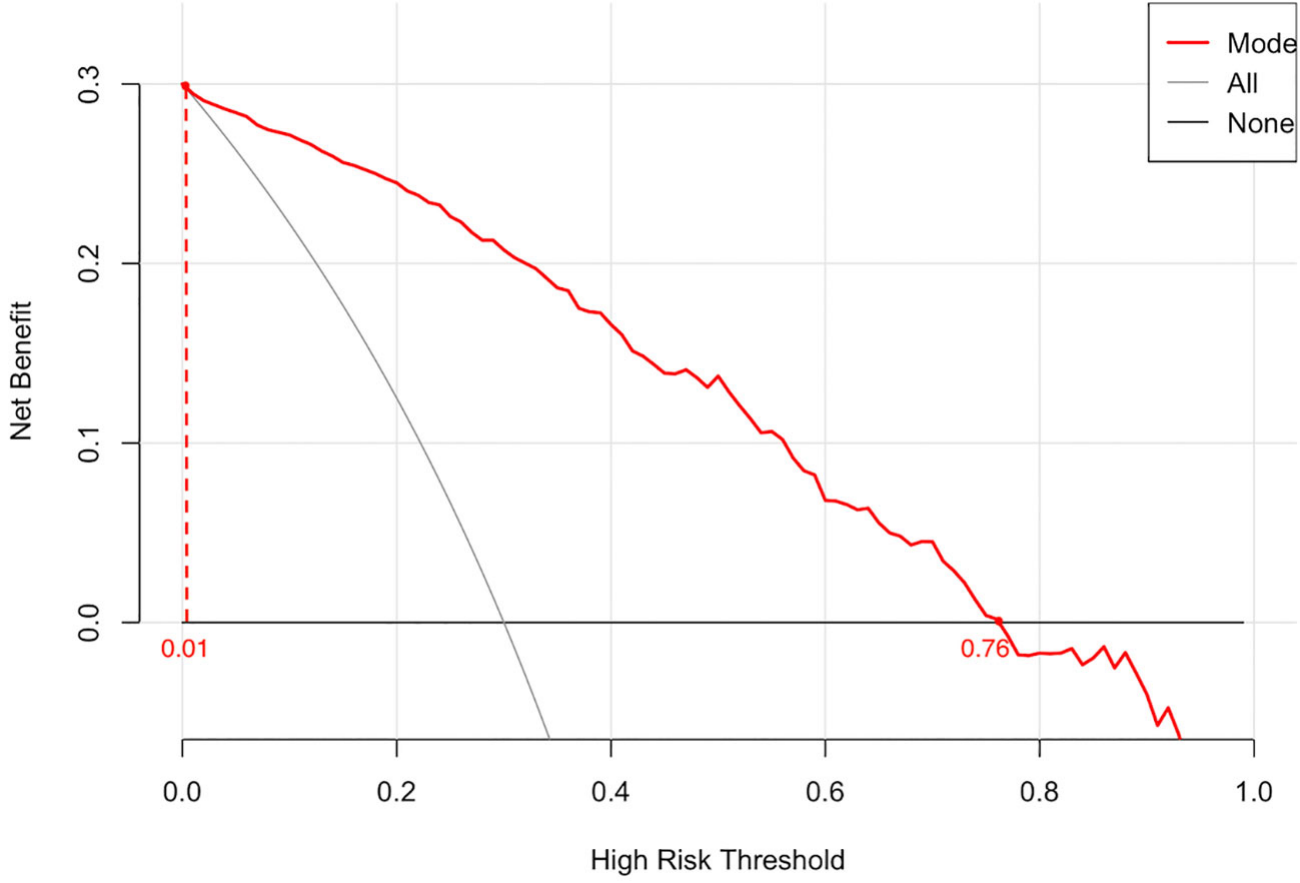
The decision curve illustrates the net clinical benefit of the model across a range of high-risk threshold probabilities. The red line represents the prediction model, while the black and gray lines correspond to the “treat-all” and “treat-none” strategies, respectively. The model demonstrates superior net benefit within the threshold range of 0.01 to 0.76, indicating promising clinical utility.

The calibration curve revealed strong agreement between predicted and observed probabilities of malignancy (**Figure 5**), with a mean absolute error of 0.038, confirming robust calibration accuracy. Decision curve analysis (DCA) demonstrated superior net clinical benefit across a threshold probability range of 0–0.8 compared to “treat-all” or “treat-none” strategies, underscoring the model’s clinical utility (**Figure 6**).

### 3.4 Web calculator prediction modeling

**Figure 7:**
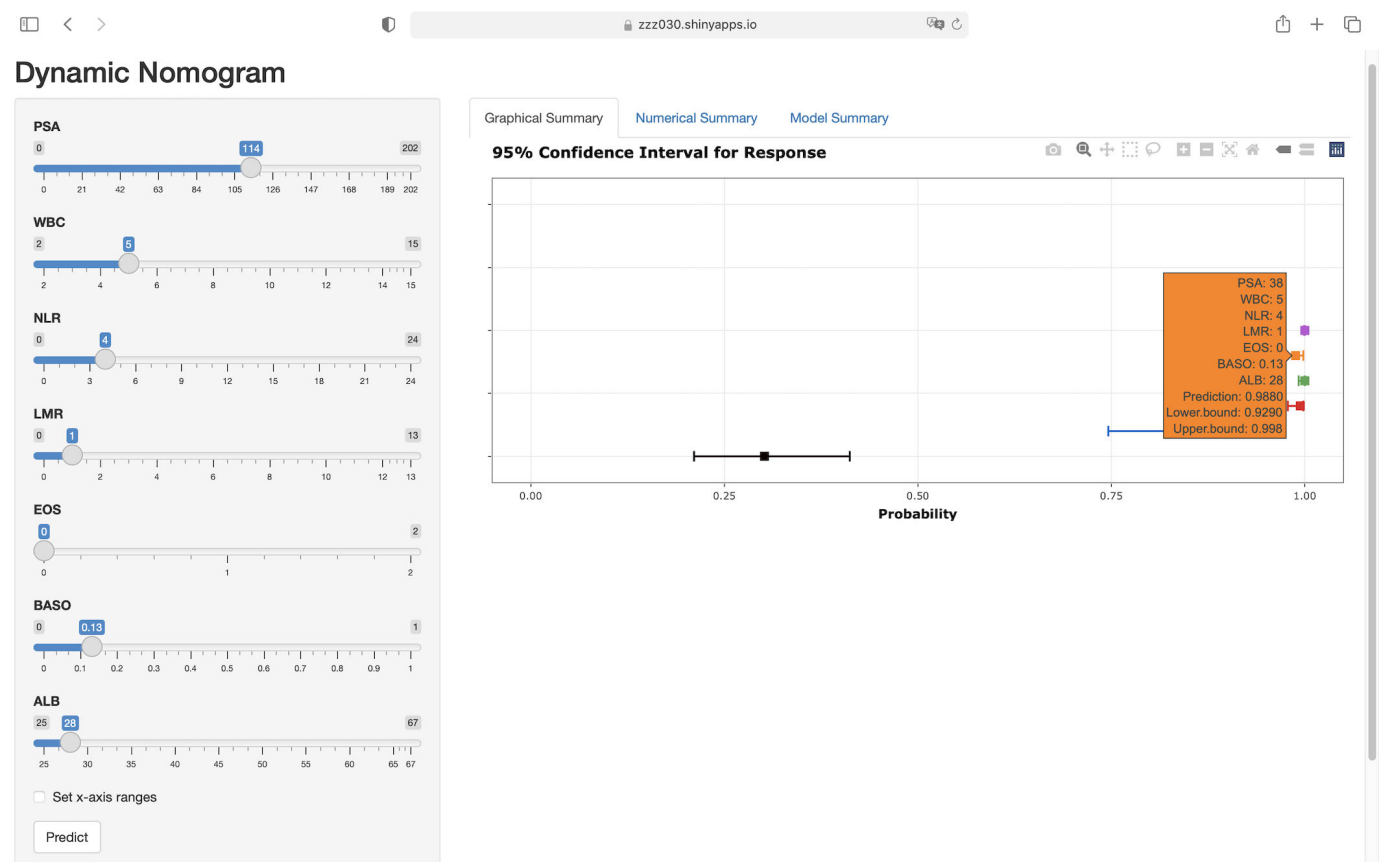
Web-based interface of the dynamic nomogram for predicting prostate malignancy risk. Users can interactively input clinical parameters to obtain individualized risk predictions. The right panel displays the predicted probability with 95% confidence intervals, enhancing interpretability and potential clinical applicability of the model. (https://zzz030.shinyapps.io/Prostate-cancer-DynNomapp/).

Based on the clinical prediction model of PSA elevated prostate benign-malignant, it was constructed in the R language environment and put on the website (https://zzz030.shinyapps.io/Prostate-cancer-DynNomapp/). The web calculator will automatically calculate the risk of PSA elevated prostate benign-malignant after inputting the clinical information of the patient. Extensive simulation testing with multiple datasets confirmed the platform’s operational stability, demonstrating robust performance of the dynamic prediction model (**Figure 7**).

## 4 Discussion

This study innovatively combined PSA with several peripheral blood markers to build a predictive model. The results demonstrated that in addition to PSA, WBC, NLR, LMR, EOS, BASO, and ALB were independent risk factors for the diagnosis of benign and malignant prostate disease in patients with elevated PSA. The constructed prediction model showed excellent performance in discriminating benign from malignant prostate disease (AUC = 0.906) and achieved a good balance between sensitivity (95.0%) and specificity (77.6%).

The inflammatory response within the tumor microenvironment is exceptionally complex, serving not only as one of the initiating factors for carcinogenesis but also participating in tumor progression, metastasis, and immune evasion (18). Immune cells such as NEUT support tumor growth and metastasis by promoting inflammation, forming neutrophil extracellular traps (NETs), and interacting with T cells (19, 20); LYM are involved in anti-tumor immunity, especially cytotoxic T cells, which inhibit tumor spread by recognizing and killing tumor cells (21); MONO differentiate into tumor-associated macrophages (TAMs) within the tumor microenvironment, which secrete immunosuppressive factors to facilitate tumor growth and metastasis (22). In recent years, increasing research has focused on alterations in inflammation-related cells and mediators during cancer initiation and progression, exploring their predictive value in tumor diagnosis and prognosis. Studies by Xinyu Yi (23) and Tian-Bao Huang (24) identified the NLR as a robust predictive biomarker for PCa, particularly in patients with PSA levels between 4 and 10 ng/mL. Further investigations by Matteo Ferro (25) and Mehmet Ilker Gokce (26) demonstrated that NLR predicts Gleason score (GS) upgrading, with higher NLR values correlating with higher-grade PCa. Our findings also validate NLR as an effective PCa predictor. However, Sat Prasad Nepal (27) reported limited diagnostic value of NLR in PCa. Emerging evidence suggests the monocyte-to-lymphocyte ratio (MLR) exhibits comparable diagnostic utility. By retrospectively analyzing data from 100 PCa patients and 103 healthy controls, Zhanping Xu et al. found that MLR was significantly elevated in patients with PCa and had high sensitivity and specificity in the diagnosis of PCa. In addition, MLR can provide higher predictive value than PSA when combined with PSA and free/total (f/tPSA) (28). Similarly, Meikai Zhu et al. identified MLR as an independent predictor for PCa and clinically significant PCa (CSPCa) in patients with PSA levels of 4–20 ng/mL (29). In an analysis of the National Health and Nutrition Examination Survey (NHANES) data by Lanyu Wang et al, MLR outperformed other inflammatory markers including NLR and PLR in PCa prediction (30). These findings are highly consistent with our LMR analysis, further validating the importance of MONO versus LYM in PCa prediction. However, compared with MLR, LMR focuses more on the dominant role of LYM, which may more directly reflect the strength of anti-tumor immune responses. In our study, we found that LMR showed some independent predictive value in the differentiation of benign and malignant prostate diseases, but the predictive ability of NLR was more significant, which may be due to the fact that NLR is sensitive to reflecting the dual signals of systemic inflammation level and immune depletion of the body and has a higher stability of the assay, whereas LMR is susceptible to the fluctuation of MONO dynamics. The diagnostic value of PLR and SII for PCa remains controversial. While some studies associate elevated PLR with higher PCa detection rates (31, 32), but others (33) did not find a significant association between the two. Similar inconsistencies exist in SII-related research (30, 34, 35). Our analysis did not identify clear diagnostic significance for PLR or SII in PCa, thus excluding them from predictive models. We hypothesize that these divergent findings may arise from variations in study population characteristics, heterogeneity in inflammatory response patterns, and inconsistent clinical risk stratification across studies. Although previous large-scale cohort studies have not demonstrated a significant association between EOS or BASO and the diagnosis of PCa (36, 37), and no existing research has incorporated them as independent variables in PCa risk prediction models, emerging evidence suggests that these two cell types may play important roles in immune regulation related to PCa progression. EOS can be recruited into the tumor microenvironment under the influence of chemokines, where they upregulate E-cadherin to inhibit cancer cell proliferation and secrete cytotoxic granules and various pro-inflammatory mediators (e.g., IL-2, IL-4, IL-5, TNF-α, TGF-β), thereby modulating T cell phenotypes and affecting tumor development (38–40). BASO can be activated by pro-inflammatory cytokines and growth factors, subsequently inducing Th2-type inflammation and M2 macrophage polarization (41, 42). They also release multiple tumor-associated mediators (e.g., IL-13, TNF-α, VEGFA, HGF), contributing to immunosuppression and tumor angiogenesis (43, 44). Therefore, based on the above biological mechanisms, this study is the first to innovatively incorporate EOS and BASO as individual indicators into the analysis, and the results ultimately confirmed their potential value in PCa risk assessment.

ALB and RBC-related parameters, as crucial biomarkers reflecting systemic physiological status, have garnered extensive attention in oncology research. These indicators mirror factors closely associated with tumorigenesis and progression, including chronic inflammatory responses, immune function alterations, and hormone level changes (45, 46). The study by Kailiang Xu et al. revealed a nonlinear relationship between ALB and PSA, particularly demonstrating an inverse correlation when serum ALB levels exceed 41 g/L. This phenomenon may be attributed to elevated ALB concentrations reducing free testosterone levels, thereby suppressing PSA expression. These findings further support ALB’s potential role in prostate disease risk assessment, with lower levels potentially indicating higher tumor burden (47). Additionally, research by Kaya C et al. demonstrated significantly lower ALB levels in PCa patients compared to those with BPH. ROC curve analysis indicated superior diagnostic performance of ALB over PSA (48). Prostate-specific membrane antigen (PSMA) probes conjugated with ALB not only enhance stability in circulation but also improve tumor accumulation, providing robust support for early PCa diagnosis, treatment monitoring, and targeted therapy (49). Other ALB-derived indices, including the albumin-to-globulin ratio (AGR) and hemoglobin-albumin-lymphocyte-platelet (HALP) score, have shown significant associations with progression-free survival (PFS) and cancer-specific survival (CSS) in metastatic PCa patients, emerging as valuable prognostic indicators (50, 51). Our findings corroborate ALB’s value as an independent predictor, with lower ALB levels significantly associated with PCa detection.

Lipid metabolism is intricately linked to tumor biology. Tumor cells provide essential lipid components for membrane biosynthesis and energy supply by enhancing the synthesis of fatty acids from scratch to meet their rapid value-added requirements (52). Lipids such as cholesterol and phospholipids not only serve as structural elements of cellular membranes but also act as signaling molecule carriers, regulating critical pathways like PI3K/AKT/mTOR to influence tumor cell proliferation and survival (53). Furthermore, tumor cells reprogram lipid metabolism to promote the secretion of immunosuppressive factors (e.g., TGF-β1), thereby inhibiting T-cell activity and facilitating immune evasion (54). In recent years, the predictive value of lipid metabolic markers in cancer risk assessment has garnered increasing attention. While Fu Feng et al. suggested that elevated TG and LDL levels may correlate with increased PCa risk (55), Hanxu Guo et al. paradoxically identified high TG as a potential protective factor (56). Conversely, Anna Ioannidou et al. found no significant association between HDL, TG concentrations and PCa risk (57). In our study, lipid metabolic parameters failed to demonstrate significant predictive value for PCa risk. This discrepancy may arise from the inherent complexity of lipid metabolic networks including dynamic regulation, individual heterogeneity, and multidimensional interactions which likely diminishes the predictive efficacy of single lipid biomarkers.

Research findings on Ca, P, and ALP in PCa also remain contentious. Sepehr Salem et al. noted that higher serum total and ionized Ca concentrations were negatively correlated with a lower risk of PCa (58). Additionally, severe hypocalcemia has been proposed as a potential indicator of bone metastasis in PCa (59). However, James Yarmolinsky et al. found a weak association between serum Ca and PCa risk by Mendelian randomization (MR) analysis and did not find a significant effect of serum Ca on advanced PCa (60). Similarly, Linshuoshuo Lv et al. employed MR to demonstrate a potential causal relationship between serum P and PCa risk, indicating a 19% increase in PCa risk per 1-standard deviation (SD) rise in serum phosphorus (61). In clinical practice, elevated ALP levels in PCa patients are commonly utilized as biomarkers for bone metastasis, reflecting tumor invasion into bone tissue and associated osteolytic activity (62). Our study found that Ca, P and ALP have limited diagnostic value in PCa risk assessment, probably because changes in these markers reflect more metabolic abnormalities or severity of bone metastases in advanced stages of the disease, related to the limited proportion of patients with advanced PCa in the sample.

Collectively, current research trends demonstrate that integrating multiple peripheral blood biomarkers with PSA levels can significantly enhance diagnostic accuracy for PCa. Our study not only enriches existing research on the combined application of blood biomarkers and PSA but also offers a comprehensive and dynamic risk assessment approach, facilitating precise early diagnosis and personalized therapeutic decision-making in clinical practice. Compared with existing tools, a study by Yuhua Huang et al. evaluated the diagnostic performance of the PHI in a Chinese population and reported an AUC of 0.766, which is notably lower than the AUC of 0.906 achieved by our model (63). Although the 4Kscore, SelectMDx, and EPI have been extensively studied internationally and demonstrated favorable predictive performance, there is a lack of systematic validation in Chinese populations. Therefore, the predictive model developed and validated in this study serves as a meaningful complement to existing mainstream models by addressing their limitations in population applicability and demonstrates notable potential and localized advantages in Chinese populations. However, this study also has several limitations: (1) The dataset originated from a single institution with regional sample characteristics. Future studies should incorporate multicenter data for external validation to improve the model’s generalizability and reliability. (2) While focusing on conventional peripheral blood biomarkers and PSA integration, future research should incorporate additional relevant markers and imaging data. MRI provides critical information such as prostate volume, lesion diameter, signal abnormalities, and extracapsular extension. Integrating these parameters could further enhance the model’s discriminative capacity in complex cases. (3) Certain blood biomarkers (e.g., PLR and SII) showed statistical associations with prostate disease risk in univariate analysis but failed to emerge as independent predictors in multivariate analysis. This discrepancy may stem from indirect correlations within patient subgroups or insufficient statistical power due to limited sample size. Larger-scale studies are warranted to validate the potential value of these markers.

## 5 Conclusion

This study developed a dynamic clinical prediction model integrating PSA and multiple peripheral blood biomarkers for distinguishing benign and malignant prostate diseases. The model serves as an effective tool for assessing malignancy risk in patients with elevated PSA levels, thereby optimizing clinical decision-making in diagnosis and treatment.

## Data Availability

The original contributions presented in the study are included in the article/supplementary material. Further inquiries can be directed to the corresponding author.
